# Species Distribution and Susceptibility to Azoles of Vaginal Yeasts Isolated Prostitutes

**DOI:** 10.1155/2007/82412

**Published:** 2007-08-20

**Authors:** Norma T. Gross, M. L. Arias, M. Moraga, Y. Baddasarow, C. Jarstrand

**Affiliations:** ^1^Centro de Investigaciones en Enfermedades Tropicales (CIET), Facultad de Microbiologia, Universidad de Costa Rica, San José 2060, Costa Rica; ^2^Departamento de Control de SIDA y Enfermedades de Transmisión Sexual, Caja Costarricense de Seguro Social, San José 1000, Costa Rica; ^3^Karolinska Institutet, Department of Laboratory Medicine F72, Karolinska University Hospital, 141 86 Stockholm, Sweden

## Abstract

*Objective*. We investigated the use of miconazole among female prostitutes in Costa Rica as well as the distribution of vaginal yeasts and the susceptibility pattern to azoles of strains obtained from this population. Our intention was to relate a frequent use of miconazole to occurrence of vaginal yeasts resistant to azoles. *Methods*. Vaginal samples were taken from 277 patients that have previously used azoles. Vaginal swabs were obtained for direct microscopy and culture. Yeast isolates were identified by germ tube test and assimilation pattern. Susceptibility testing was determined using a tablet diffusion method. *Results*. The number of clinical *Candida* isolates (one from each patient) was 57 (20.6%). *C. albicans* was the predominant species (70%), followed by *C. parapsilosis* (12%), *C. tropicalis* (5.3%), *C. glabrata* and *C. famata* (3.5% each), *C. krusei*, *C. inconspicua* and *C. guilliermondii* (1.7% each). The majority of vaginal *Candida* isolates were susceptible to ketoconazole (91%), fluconazole (96.5%), and itraconazole (98%). A lower susceptibility of some isolates to miconazole (63%) was observed as compared to the other azoles tested. Moreover, the strains, nonsusceptible to miconazole, were more often obtained from patients that have used this antifungal at least four times within the last year before taking the samples as compared to those with three or less treatments (P<.01). *Conclusion*. An indiscriminate use of miconazole, such as that observed among female prostitutes in Costa Rica, results in a reduced susceptibility of vaginal yeasts to miconazole but not to other azoles.

## 1. INTRODUCTION

*Candida* may be either a commensal or a pathogen of the vagina, a fact which indicates that changes in the vaginal microenvironment are generally necessary for *Candida* to induce pathological changes associated with clinical symptoms [1]. Vulvovaginal candidosis (VVC) is a common cause of vaginal discharge, soreness, vulval burning, dysuria and local pruritus in women. *C. albicans* is the most common etiological agent of VVC, but other species such as *C. glabrata*, *C. krusei*, and *C. tropicalis* are also encountered [1–3].

Depending on the age, locality, and social economical status, the frequency of vaginal yeast isolates has been reported to be between 5% and 48.4% in healthy nonpregnant women [4–6]. During pregnancy, an increase of 7.8% and 67.8% of yeast vaginal isolates has been reported [7, 8]. A study in Jordan revealed a different distribution of strains of *Candida* spp. among pregnant, infertile, and (nonpregnant, fertile) healthy controls [6].

Azoles are antifungal drugs frequently used for treatment of VVC. There is evidence, however, of an increased azole resistance among isolates of *Candida* spp. isolated from patients with VVC [9, 10]. Non-*C. albicans* are generally more resistant to azoles than are *C. albicans* strains [11]. Particularly in patients with VVC, several authors have found an increased prevalence of *C. glabrata* due to a selection mechanism related to the frequent use of imidazoles (ketoconazole and miconazole) [5, 12, 13]. VVC caused by a *C. albicans* strain, resistant to fluconazole after azole treatment, has been reported [14]. Several authors using genetic analysis have shown that the most common factor seen in recurrent VVC is the development of resistance during treatment [15, 16].

In the present work, we wanted to study the occurrence of vaginal yeasts, their species distribution, and the susceptibility pattern to azoles of the strains obtained from female prostitutes who attended at a public clinic in San José, Costa Rica. Since an indiscriminate use of self-prescribed azoles is frequent in this population, our aim of this study was also to determine if this behavior might cause resistance of infecting yeasts to these antifungal drugs.

## 2. SUBJECTS AND METHODS

### 2.1. Patients

Vaginal samples were collected during 24 months from 277 prostitutes examined at the Control Center of AIDS and Sexual Transmitted Diseases, Caja Costarricence del Seguro Social, San José, Costa Rica. The study included patients with or without clinical symptoms of vulvovaginitis (pruritus, dysuria, or vulval burning). All patients had previously taken miconazole (≥1−≤3 and ≥4−≤10 times) within the last year before the clinical samples were taken. Each patient completed a questionnaire about age, history of diabetes, and use of contraceptives, vaginal douche, and antimicrobial agents. Vulvovaginal symptoms and findings at the gynecological examination such as type of secretion, vulval oedema, and vulval erythema were recorded.

### 2.2. Sampling procedure

Two sterile swabs were obtained from each patient. One of the swabs was inoculated into Sabouraud dextrose agar containing penicillin (0.01 g mL^−1^) and streptomycin (0.03 g mL^−1^). The plates were then incubated at room temperature (25–30°C) for 5 days. The second swab was first streaked into a glass slide for Gram preparation, and then placed in a tube containing 0.5 mL of sterile 0.85% saline solution. A wet saline mount and a 20% potassium hydroxide preparation of the sample were then mounted on glass slides for the observation of trichomonas and yeast cells and/or hyphae in a light microscope using low magnification.

### 2.3. Identification of the yeast isolates

Yeast cultures were identified as *C. albicans* by development of germ tubes in human serum. Germ tube-negative isolates were identified by their assimilation pattern with the ID 32C identification system for yeasts (BioMerieux, France). The identified yeast isolates were maintained in distilled water at 5°C.

### 2.4. Susceptibility testing

The tablet diffusion method (Neo-Sensitabs, ROSCO Diagnostica, Taastrup, Denmark [17, 18]) was performed for all isolates according to the manufacturer's instructions. An inoculum containing approximately $5\times 10^{5}$ colony forming units (CFU) mL^−1^ (McFarland 0.5, diluted 1:1 with 0.85% saline solution or diluted 1:10 in the case of *C. krusei*) was prepared. One mL of this suspension was poured onto the agar surface (flooding) and excess liquid was removed immediately with a pipette. Thereafter, the open plates were dried at 37°C for 15 minutes and the tablets were placed on the agar surface. The azoles tested were miconazole, ketoconazole, fluconazole, and itraconazole. Plates were incubated at 37°C for 18 hours and the inhibition zones were measured. *C. albicans* ATCC 64548 and ATCC 64550 were included as the quality controls for susceptibility testing. Isolates were classified as susceptible, intermediate, and resistant according to the manufacturer's recommendations as follows: for miconazole, ketoconazole, and fluconazole, susceptible = ≥ 20 mm, intermediate = 12–19 mm, resistant = ≤ 11 mm and for itraconazole, susceptible = ≥ 15 mm, intermediate = 10–14 mm, resistant = no zone.

Yeast isolates classified by the tablet diffusion method as intermediate or resistant to fluconazole were tested according to the microdilution method proposed by the European Committee on Antibiotic Susceptibility (EUCAST) [19]. Fluconazole powder was provided by Pfizer Inc. (Stockholm, Sweden). Results for miconazole were only obtained by the tablet diffusion method since susceptibility testing to this antifungal has not yet been standardized by either the M-27 A2 reference method of NCCLS (now CLSI, Clinical Laboratory Standard Institute) [20] or the EUCAST.

## 3. RESULTS

### 3.1. Patients

Of the 277 patients examined, 106 were symptomatic (38%) and 171 were asymptomatic (62%). Among patients with symptoms, 48% had only one of the symptoms (itching, vulvar burning, or dysuria), 32% and 20% had two and three symptoms, respectively (Table 1). The age range of the patients was from 18 to 50 years and the mean age was 30 years. Only 12 patients (4.3%) were taking antibacterial treatment. Two patients (0.72%) were diabetic. Vaginal douche was a common practice in 122 (44%) of the patients. For fertility protection, some of the patients used more than one method with 105 patients taking estrogens, 16 using intrauterine devices, 118 using condoms, 32 were protected by salpingectomy and 70 unprotected.

### 3.2. Identification of the yeast isolates

The number of patients with clinical *Candida* isolates (each patient was counted only once) was 57 (20.6%). Of these isolates 36 were found among patients with symptoms and 21 from those who were asymptomatic (Table 1). *C. albicans* was the predominant species (70%), followed by *C. parapsilosis* (12%). The identity of all *Candida* species isolated is given in detail in Table 2. It was found that only 36 (34%) out of 106 vaginal samples obtained from patients with vulvovaginitis were culture positive for *Candida* spp. Thirty-one patients with symptoms had negative yeast cultures and reported the use of condom. Twenty-nine patients with symptoms had negative yeast cultures and reported the use of acetic acid in vaginal douche. *Trichomonas vaginalis* and Gram-negative pleomorphic bacilli were reported in 6 and 2 patients with negative yeast cultures, respectively.

### 3.3. Susceptibility testing

Among the different species of *Candida*, resistance to fluconazole (MIC, >64 *μ*g/mL, EUCAST method) was observed with one isolate of *C. krusei* and another one of *C. glabrata*. The *C. krusei* isolate had zone diameters for fluconazole, ketoconazole, and miconazole ≤ 11 mm (resistant), and 22 mm for itraconazole (susceptible). The *C. glabrata* isolate had zone diameters for fluconazole, ketoconazole, and miconazole ≤ 11 mm and no zone for itraconazole (resistant to all azoles tested). Three isolates intermediate to ketoconazole were observed. The isolated strains were *C. albicans* (2) and *C. glabrata* (1). As to miconazole, resistant isolates were identified as *C. parapsilosis* (2), *C. albicans* (1), and *C. glabrata* (1). The strains intermediate to miconazole were *C. albicans* (9), *C. parapsilosis* (3), *C. tropicalis* (2), *C. glabrata* (1), and *C. inconspicua* (1).

Tables 2 and 3 summarize the in vitro susceptibility of 57 *Candida* strains to fluconazole, itraconazole, ketoconazole, and miconazole as determined by a tablet diffusion test. Table 1 shows that miconazole resistant strains were found to a high degree among asymptomatic as well as among patients with symptoms. In Table 3, the isolates were divided into two groups, those from women treated with miconazole ≤3 and those treated ≥4 times during the last year before taking the samples. When the two groups containing 22 (≤3) and 35 isolates (≥4) were compared, 3 (13.6%) and 18 isolates (51.4%), respectively, were nonsusceptible to miconazole. The difference is significant (P<.01 Fisher's exact test [21]).

## 4. DISCUSSION

In Costa Rica, the use of miconazole is very frequent among prostitutes as a treatment for vaginal candidasis. It is also a common belief among this group of women that the constant use of antifungal will prevent yeast vaginal infection. The present study was therefore undertaken to examine the occurrence and species distribution of vaginal yeasts and to evaluate susceptibility of yeast isolates recovered from vaginal samples to miconazole. Susceptibility testing to ketoconazole, fluconazole, and itraconazole was also performed. In contrast to other azoles, miconazole possesses two mechanisms of action and yeasts are most often found to remain susceptible even after repeated exposures [22]. Our study shows however that after more than three treatment periods miconazole resistant strains are frequently found.

Our particular patient population always chose miconazole and used it even without clinical symptoms of vulvovaginitis. The misuse of this drug is reflected by the fact that resistant Candida strains were found to a high degree among asymptomatic patients (Table 1). The reasons for the choice of miconazole are that this drug, in Costa Rica, is available over-the-counter and that it is less expensive than other azoles. A control group, that is patients that had taken repeated treatments (more than three times) of azoles other than miconazole, was not possible to obtain. However, our patients with three treatments or less can be considered as controls to those with four or more treatments. A significantly higher number of nonsusceptible yeast isolates were found in this latter group than among the controls (Table 3).

A previous study in female sexual workers revealed that the most frequently isolated vaginal yeast was *C. albicans*, followed by *C. glabrata* [10]. In the general population, several studies also indicate that*C. albicans* predominates over other species. *C. glabrata* is the second one most common, but other species such as *C. krusei*, *C. parapsilosis*, *C. tropicalis *, *C. guilliermondii*, *C. kefyr* are also encountered [2, 3, 6]. In accordance with these previous authors, in our study *C. albicans* was the dominant vaginal isolate. However, *C. parapsilosis* was the second one most common, followed by *C. tropicalis* and then by *C. glabrata.*

Clinical symptoms such as itching, vulvar burning, and dysuria are not always indicative of VVC [1], which is in agreement with our results. Among possible explanations for these findings are contact dermatitis or allergic reactions probably related to the use of condom or other devices such as acetic acid used in vaginal douche. Other causative agents could be involved, such as *Trichomonas vaginalis* or Gram-negative pleomorphic bacilli. These observations are important to consider for adequate treatment of vulvovaginitis.

In a previous in vitro susceptibility study of vaginal isolates in Jordan, miconazole nitrate was less effective as compared to amphotericin B, nystatin, and chlorhexidine. In the study, however, no other azoles were examined [6]. The susceptibility to fluconazole of *C. albicans* and *C. glabrata* isolated from female prostitutes with vulvovaginitis was evaluated in another study [10]. These authors reported a greater susceptibility to fluconazole for *C. albicans* compared to *C. glabrata*. They isolated one (4.3%) resistant strain of *C. glabrata* and four dose-dependent susceptible strains (17.3%).

The reference method for susceptibility testing to azoles (fluconazole, ketoconazole, and itraconazole) is the document M27-A2, proposed by CLSI [20]. However, susceptibility testing to miconazole has not been standardized. The method used in the present study, the tablet diffusion (Neo-sensitabs), is an alternative method which offers susceptibility testing to miconazole with interpretation of the zone diameters for local infections. Thus, the results of our study were analyzed in accordance with this method.

Concerning the susceptibility of the vaginal isolates to azoles, our data indicate that the majority of the isolates were susceptible to the azoles evaluated. Resistance to fluconazole was observed with one isolate of *C. krusei*. The isolate was also resistant to ketoconazole and miconazole, but not to itraconazole. It is well-established that *C. krusei* is intrinsically resistant to fluconazole and ketoconazole [23, 24]. As to itraconazole, *C. krusei* is usually susceptible [21]. One isolate of *C. glabrata* also exhibited resistance to fluconazole and the rest of the azoles tested. It is known that *C. glabrata* can develop resistance to fluconazole as a primary and secondary mechanism [24].

In the present study, miconazole which was the azole used by all patients showed the lowest percentage of susceptibility (63%). It is also of interest that 9% of the isolates were resistant and 28% were intermediate to miconazole. Further, most of the nonsusceptible strains to miconazole were obtained from women that had been taking this antifungal four or more times during the last year before taking the clinical samples. In our study, there was only one *C. albicans* resistant isolate. However, among the isolates classified as intermediate, there were 9 *C. albicans*. Our finding is in agreement with previous observations that the majority of yeast isolates that develop resistance while on treatment are *C. albicans* [25–28]. Taking these observations into account, it is recommended to advice this group of women not to take antimicrobials indiscriminatively.

In conclusion, the great majority of vaginal *Candida* isolates exhibited susceptibility to ketoconazole, fluconazole, and itraconazole. However, a tendency for lower susceptibility of some isolates to miconazole was observed as compared to the other azoles tested, a finding that could be related to the abuse of miconazole treatment by female prostitutes in Costa Rica.

## Figures and Tables

**Table 1:** Candida isolates in patients without (n=171) or with different symptoms of vulvovaginitis (n=106) all with previous miconazole treatment.

Symptoms	Number of patients	Number of Candida isolates	Number of miconazole nonsusceptible strains
Itching	45	—	—
Vulval burning or dysuria	6	—	—
Itching, vulval burning, and dyuria	21	—	—
Summary of patients with symptoms	106	36	6
Patients without symptoms	171	21	13

**Table 2:** Azole nonsusceptibility of Candida isolates from vagina of women with or without vulvovaginitis and with previous miconazole treatment. Total number of isolates = 57.

Yeast	Number of	Fluconazole	Itraconazole	Ketoconazole	Miconazole
	isolates	nonsusceptible	nonsusceptible	nonsusceptible	nonsusceptible
*C. albicans*	40	—	—	—	10
*C. parapsilosis*	7	—	—	—	5
*C. tropicalis*	3	—	—	—	2
*C. glabrata*	2	1	1	2	2
*C. famata*	2	—	—	—	—
*C. krusei*	1	1	—	1	1
*C. inconspicua*	1	—	—	—	1
*C. guillermondii*	1	—	—	—	—

Total number	57	2	1	3	21

**Table 3:** Azoles susceptibility pattern of *Candida* spp. isolated from vagina of women with or without vulvovaginitis with previous miconazole treatment (≤3 or ≥4 times). Total number of isolates = 57 (consisting of 22 and 35 isolates from women taken miconazole ≤3 and ≥4 times, respectively, during the last year before taking the samples). When the two groups (≤3 and ≥4) were compared as to miconazole nonsusceptibility, the *P* value was < .01.

	Resistant		Intermediate		Nonsusceptible (resistant + intermediate)		Susceptible	
	≤3	≥4	≤3	≥4	≤3	≥4	≤3	≥4
Fluconazole	1	1	0	0	1	1	20	35
Itraconazole	1	0	0	0	1	0	20	36
Ketoconazole	1	1	2	1	3	2	18	34
Miconazole	1	4	2	14	3	18	19	17
